# Nutritional supplementation: the additional costs of managing children infected with HIV in resource-constrained settings

**DOI:** 10.1111/tmi.12006

**Published:** 2012-10-29

**Authors:** G Cobb, R M Bland

**Affiliations:** 1 Africa Centre for Health and Populations Studies, University of KwaZulu-NatalKwaZulu-Natal, South Africa; 2 School of Medicine, University of GlasgowGlasgow, UK

**Keywords:** paediatric, HIV, nutrition, severe acute malnutrition, undernutrition, nutritional supplements, World Health Organization Nutritional Care Plans

## Abstract

**Objective:**

To explore the financial implications of applying the WHO guidelines for the nutritional management of HIV-infected children in a rural South African HIV programme.

**Methods:**

WHO guidelines describe Nutritional Care Plans (NCPs) for three categories of HIV-infected children: NCP-A: growing adequately; NCP-B: weight-for-age z-score (WAZ) ≤−2 but no evidence of severe acute malnutrition (SAM), confirmed weight loss/growth curve flattening, or condition with increased nutritional needs (e.g. tuberculosis); NCP-C: SAM. In resource-constrained settings, children requiring NCP-B or NCP-C usually need supplementation to achieve the additional energy recommendation. We estimated the proportion of children initiating antiretroviral treatment (ART) in the Hlabisa HIV Programme who would have been eligible for supplementation in 2010. The cost of supplying 26-weeks supplementation as a proportion of the cost of supplying ART to the same group was calculated.

**Results:**

A total of 251 children aged 6 months to 14 years initiated ART. Eighty-eight required 6-month NCP-B, including 41 with a WAZ ≤−2 (no evidence of SAM) and 47 with a WAZ >−2 with co-existent morbidities including tuberculosis. Additionally, 25 children had SAM and required 10-weeks NCP-C followed by 16-weeks NCP-B. Thus, 113 of 251 (45%) children were eligible for nutritional supplementation at an estimated overall cost of $11 136, using 2010 exchange rates. These costs are an estimated additional 11.6% to that of supplying 26-week ART to the 251 children initiated.

**Conclusions:**

It is essential to address nutritional needs of HIV-infected children to optimise their health outcomes. Nutritional supplementation should be integral to, and budgeted for, in HIV programmes.

## Introduction

Childhood malnutrition is associated with increased mortality and morbidity irrespective of HIV status. Up to 30% of deaths in children aged under 5 years are attributable to undernutrition defined as a weight-for-age z-score (WAZ) <−1 (Black *et al*. 2008). The prevalence of stunting and undernutrition increases inversely with household socio-economic status (SES), whilst stunting is synonymous with chronic undernutrition and an indicator of social deprivation (WHO 1986).

Up to 50% of children initiated on antiretroviral treatment (ART) in African HIV treatment programmes are reported to be undernourished (Bolton-Moore *et al*. 2007), and severe malnutrition at initiation of ART is a recognised risk factor for mortality in HIV-infected children (Sutcliffe *et al*. 2008), especially when co-infected with HIV and TB (Hesseling *et al*. 2005).

Both antiretroviral treatment (ART) and good nutrition are important for the management of HIV-infected, malnourished children. Improvements in anthropometric measurements have been demonstrated in undernourished HIV-infected children following the initiation of ART irrespective of baseline nutritional status or the provision of nutritional supplements (Davies *et al*. 2009; Sutcliffe *et al*. 2011). Those who are severely malnourished demonstrate similar improvements in anthropometric measurements following the initiation of ART and nutritional therapy as HIV-uninfected children with SAM but have a higher initial mortality rate (Fergusson *et al*. 2009). In a recent study from Malawi, prompt initiation of ART within 3 weeks of starting therapeutic feeding was associated with better nutritional outcomes compared with those who started ART after this time (Kim *et al*. 2012).

The WHO published ‘Guidelines for an Integrated Approach to the Nutritional Care of HIV-Infected Children (the ‘Guidelines’) (WHO 2009). The Guidelines provide a structured ten-step plan for the nutritional management of HIV-infected children aged 6 months to 14 years and can be applied to both inpatient and outpatient settings. Three ‘Nutritional Care Plans’ (NCP-A, NCP-B and NCP-C) are recommended depending on the child's weight status (based on anthropometric measurements) and presence of comorbidities (see Box 1). For each NCP, the specific additional daily calorific intake for a child in that category is given (Box 1).

**Box 1: WHO Nutritional Care Plans**

- **NCP-A** for children who are growing appropriately as determined using WHO Child Growth Standards (The WHO Child Growth Standards, 2006).
  Children require an additional 10% of energy based on their actual weight, usually provided as part of the diet and not nutritional supplements
- **NCP-B** for children with poor weight gain or increased nutritional needs including reported weight loss, very low weight (WAZ <−3), underweight (WAZ <−2), confirmed weight loss (>5%) since the last visit, or growth curve flattening.
  Morbidities with increased nutritional requirements include chronic lung disease, TB, persistent diarrhoea, or other chronic opportunistic infection or malignancy (this will include most children who have stage 3 or 4 disease).
  These children require an additional 20–30% energy per day based on their age.
- **NCP-C** for children with severe malnutrition including signs of severe visible wasting, oedema present in both feet, WHZ <−3, or MUAC <115 mm for children aged 6–60 months, 129 mm in children 5–9 years or 160 mm in children 10–14 years.
  These children require an additional 50–100% energy per day in the form of therapeutic feeds with total daily energy calculated on their weight and age.

Whilst the development of these Guidelines is a significant advance in the nutritional management of HIV-infected children, the financial cost of supplying these additional calories with macronutrient supplements in rural HIV programmes needs to be explored. We investigate the proportion of children in a rural South African HIV programme who would be eligible for supplementation at the time of ART initiation, the proportion within each category of NCP and the costs of purchasing the appropriate NCP with locally available nutritional supplements.

## Methods

### The setting

The Hlabisa HIV Treatment and Care Programme (the ‘HIV Programme’) is a partnership between the South African Department of Health (DoH) and the Africa Centre for Health and Population Studies (http://www.africacentre.ac.za) with additional funding provided by PEPFAR (Houlihan *et al*. 2010a). This rural subdistrict has an estimated adult HIV prevalence of 22% (Barnighausen *et al*. 2008) and a TB notification rate of 1700 per 100 000 population (Wallrauch *et al*. 2008). In 2008, two-thirds of the population survived on <$2 per day (Muhawava 2008).

### Definitions of undernutrition

WHO Growth Standards (children aged up to 5 years) and References (children aged from 5–14 years) were used to define children with undernutrition World Health Organization, United Nation's Children's Fund (2009). Severe acute malnutrition (SAM) was defined as a WHZ of <−3, a mid-upper arm circumference (MUAC) less than the reference range for age (<115 mm 6–60 months, <129 mm 5–9 years and <160 mm 10–14 years), or on clinical findings of visible wasting or bilateral pedal oedema. Children with SAM were eligible for NCP-C management (nutritional stabilisation).

A weight-for-age z-score or WHZ ≤2 but with no signs of SAM indicated moderate undernutrition. Children with moderate undernutrition, or with flattening of their growth curve, or any comorbidity requiring additional daily energy intake (e.g. TB or persistent diarrhoea), required NCP-B (nutritional rehabilitation) (see Box 1).

### Healthcare provision

The decentralised HIV Programme supports 17 nurse-led primary healthcare clinics (PHCs) with a district hospital providing inpatient care (Houlihan *et al*. 2010a). At the time of this study, physicians assessed all HIV-infected children at clinics for their eligibility to initiate ART in accordance with the National Department of Health Guidelines based on the WHO 2006 paediatric ART guidelines (South African Dept of Health 2010). Nutritional status was evaluated for all children at initial assessment using WAZ and physical examination for signs of SAM. Height and MUAC were not routinely assessed in the programme because of a lack of stadiometers and tape measures and the large patient workload in the clinics (personal communication Kevi Naidu, Africa Centre).

### Data management

Patient data, recorded in paper-based clinic files at initiation of ART, are entered into a secure electronic programme database housed at the Africa Centre (Houlihan *et al*. 2010a). The database was accessed to obtain data for all children aged 6 months to 14 years at initiation of ART during the period 1 January to 31 December 2010. Variables of interest included weight-for-age, prevalent TB and WHO Clinical Stage. Where data were missing in the database, clinic files were reviewed and missing data retrieved where available.

### Nutritional supplements

The proportion of children who were eligible for NCP-B or NCP-C at ART initiation was determined using the criteria in the Guidelines (Box 1). Commercial supplements available in South Africa, and to our programme, included Future Life Porridge® (Futurelife, PO Box 1035, New Germany, 3620, South Africa), used for nutritional rehabilitation (NCP-B), and Sibusiso®, a ‘ready-to-use food’ (RTUF) (Manary *et al*. 2004), used only for the stabilisation phase of SAM (NCP-C). Sibusiso is stored at room temperature and does not need to be prepared or reconstituted (Gift of the Givers Foundation, Pietermaritzburg, South Africa). No generic supplements were available.

### Calculating energy requirements

The daily additional energy requirements for the children who would have been eligible for supplementation according to NCP-B or NCP-C were calculated. We used the upper recommended additional daily calorific intake (30% additional intake for NCP-B and 100% for NCP-C) to compensate for the high level of household food insecurity and poverty in the area (Table 1). These calculations provided an overall energy requirement for the groups and were used to determine the quantity of supplements required for a 26-week period per child (see rationale for duration of supplementation below).

**Table 1:** Daily additional calorific requirement by age, weight and care plan (adapted from WHO chart booklet pages 7 and 8)

Age group	NCP-B daily energy requirements as supplementation, kcal	NCP-C total daily energy requirements*, kcal/kg
6–11 months	120–150	150–220
12–23 months	160–190	150–220
2–5 years	200–280	150–220
6–9 years	260–380	75–100
10–14 years	340–400	60–90

* 50% of this total daily requirement is supplied through supplementation with the other 50% obtained through household provision and is for 10 weeks only before reducing supplementation from NCP-C to NCP-B.

The cost of purchasing Future Life Porridge for all children eligible for NCP-B was determined using the unit cost per child by age group multiplied by the number of children within that age group. Costs for all age groups were summed to provide the total cost for the cohort eligible for NCP-B.

Calculations for NCP-C provide the *total* daily energy requirement as previously calculated by the WHO (WHO 1999) and include calories obtained from both nutritional supplements *and* household food supply (Table 1). It is recommended that half of this total requirement is provided through nutritional supplementation for this NCP. Individual calculations were necessary to determine total energy requirements per child as these depend on the child's weight and age and the stage of supplementation (see below). These calculations were then summed to provide the cost of purchasing NCP-C for all children with SAM.

### Time period for supplementation

A 26-week time frame was deemed an appropriate period of supplementation for all undernourished children for a number of reasons. This period is recommended by the WHO for follow-up of children with SAM irrespective of HIV status (WHO 1999), it allows for recovery from conditions associated with increased energy requirements (including 6-month TB treatment), it allows time to achieve standardised exit criteria (highlighted below) and provides some compensation for household food insecurity and inadequate provision of calories at the household level. In all cases, the Guidelines recommend the continued provision of supplements where food security is compromised.

Exit criteria are provided for discontinuing or reducing the amount of nutritional supplements provided. These recommend that children requiring NCP-B who have achieved a WAZ or WHZ >−1 AND gained weight over at least two consecutive clinic visits over approximately 30 days AND shown resolution of any condition with increased energy needs, have demonstrated an adequate response to nutritional therapy and can be moved to NCP-A (at which point supplementation is discontinued).

Exit criteria for downgrading from NCP-C to NCP-B in SAM includes loss of oedema AND recovery of appetite AND achievement of WHZ ≥−1, OR 15% weight gain after loss of oedema (OR gaining at least 5–15 g/kg/day in hospital or 5 g/kg/day in the community if no height recorded). The Guidelines recommend SAM children receive 6- to 10-weeks nutritional stabilisation (NCP-C) followed by 16- to 20-weeks nutritional rehabilitation (NCP-B). Our estimates were based on 10 weeks of NCP-C using Sibusiso followed by 16-weeks NCP-B using Future Life Porridge.

### Calculating the costs of nutritional supplementation for the cohort

Purchase costs for Future Life Porridge and Sibusiso at 2010 bulk prices charged to the HIV Programme by the manufacturers were used to calculate the cost of providing supplements to meet the additional daily calorific requirement per child and per NCP for 26 weeks. Costs calculated in South African Rand (ZAR) were converted into US dollars (USD) using the 2010 mid-year exchange rate (02 July 2010: 1ZAR = 0.129USD). The cost per kcal (ZAR/kcal) was determined from the cost of the supply unit (500 g Sibusiso or 500 g Porridge) and the number of calories this contained (calories/unit) (Tables 2 and 3).

**Table 2:** Estimated cost of providing supplements (Future life porridge) for 26 weeks to the cohort requiring NCP-B only

Age group	Unit costs (Per Child)			Estimated total costs for cohort (26 weeks)	
	
	Additional calories per child per day (kcal/day)	Total calories for 26 weeks (kcal)	Cost of supplements (ZAR)	Number of children (88)	Cost of supplying NCP-B by age group
6–11 months	150	27 300	246	9	2214
12–23 months	190	34 580	311	12	3732
2–5 years	280	50 960	459	19	8721
6–9 years	380	69 160	622	21	13 062
10–14 years	400	72 800	655	27	17 685
Estimated Total cost for cohort					ZAR45,414
Estimated Total cost per child					ZAR516

Conversion rate ZAR to USD using 2010 mid-year exchange rate (02 July 2010: 1ZAR = 0.129USD). The cost per kcal for Future Life Porridge = ZAR0.009/kcal.

**Table 3:** Costs of providing supplements for 26 weeks to the cohort with SAM (10-weeks NCP-C with Sibusiso and 16-weeks NCP-B with Future Life Porridge)

Age of child	Number of children (25)	Estimated total calories required 10-weeks NCP-C (kcal)	Estimated total cost for 10-weeks NCP-C (ZAR)	Estimated total calories required 16-weeks NCP-B (kcal)	Estimated total cost for 16-weeks NCP-B (ZAR)	Estimated total cost for cohort (26 Weeks) (ZAR)
6–11 months	4*	168 630	3541	85 500	770	4311
12–24 months	5	311 080	6533	106 875	962	7495
2–5 years	4	307 230	6452	126 000	1134	7586
6–9 years	3	142 800	2999	128,250	1154	4153
10–14 years	9	653 310	13 720	405 000	3645	17 365
Estimated total for cohort		1 583 050	33 245	851 625	7 665	ZAR40,910
Estimated total per child						ZAR1,636

* One child aged 6–12 months did not have a weight recorded at initiation but was documented as having SAM by WHO staging. A weight of 6.5 kg was used for this child as the heaviest weight giving a WAZ <−3 although the child's actual weight is likely to be less and thus the calculation for individual NCP-C an overestimate but unlikely to significantly affect the overall cost of supplying supplements to this cohort.

Conversion rate ZAR to USD using 2010 mid-year exchange rate (02 July 2010: 1ZAR = 0.129USD).

Unit cost (ZAR) of Sibusiso = 0.021/kcal and of Future Life Porridge = 0.009/kcal.

### Costs of supplying antiretroviral drugs for 6 months to the same group of children

As a point of reference, the cost of providing 26-week first-line ART for the same children was calculated using liquid formulations for children ≤3 years and tablet preparations for those >3 years and the mean weight for children in these age groups. South African DOH drug regimens were used (abacavir, lamivudine and lopinavir/ritonavir for children ≤3 years, and abacavir, lamivudine and efavirenz for children >3 years).

### Ethics

Ethics approval was obtained from the Biomedical Ethics Committee of the University of KwaZulu-Natal for the retrospective analysis of anonymised data from the HIV Treatment and Care Programme (BE066/07); approval was also granted by the Research Office of the KwaZulu-Natal Department of Health.

## Results

**Figure 1:**
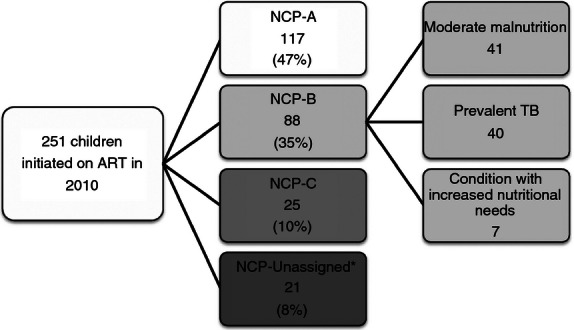
Number of children initiating ART by nutritional care plan. *Twenty-one children were unassigned because of missing data.

In 2010, 251 children aged 6 months to 14 years initiated ART in the Programme. Of these, 113 (45%) would have been eligible for nutritional supplementation according to the Guidelines of whom 88 (78%) would have required 6-month NCP-B (40 with prevalent TB, 41 with moderate malnutrition and 7 with a condition with increased nutritional needs); 25 (22%) with SAM (4 of whom also had prevalent tuberculosis) would have required NCP-C for 10 weeks followed by NCP-B for 16 weeks (Figure 1). In total, 44 of 251 (17.5%) children had prevalent TB at initiation and an additional 24 had a previous history of TB. Thus, 69 of 251 (27.5%) children initiating ART had a history of TB disease.

For 21 (8%) children, it was not possible to classify which NCP was required from the data available (because of lack of clinical data regarding WHO staging and presence of co-existent disease such as TB), and they were classified as ‘unassigned’.

The cost of supplying each additional kcal with nutritional supplements equated to 0.021 ZAR/kcal for Sibusiso and 0.009 ZAR/kcal for Future Life Porridge. Costs of providing 26-week NCP-B (Future Life Porridge) equated to ZAR516 per child and ZAR 45,414 ($5858) for all 88 children requiring NCP-B (Table 2) and ZAR1,636 per child or ZAR40,910 ($5277) for all 25 children with SAM who were provided with 10-weeks NCP-C (Sibusiso) and 16-weeks NCP-B (Future Life Porridge) (Table 3). Thus, the cost of 26-weeks supplementation for the entire group of 113 children requiring supplementation was ZAR86,324 ($11 135).

Of the 21 children with missing clinical staging or evidence of TB at initiation, 20 had a WAZ >−2 (thus unlikely to have SAM unless markedly oedematous), a further child did not have any weight or clinical details recorded. If we assume that all these children had a stage 3 or 4 disease requiring supplementation with NCP-B, then the additional cost of supplementing these children determined by their age would be ZAR12,449 ($1606). This estimation provides an upper limit of ZAR98,773 ($12 742) for purchasing supplements for all children initiated during the year period.

The mean weight of all children aged ≤3 years initiating ART was 9.8 kg (92 children) and for children >3 years was 21.6 kg (159 children). The total estimated cost of supplying first-line ART for 26 weeks to all 251 children was ZAR745,637 ($96 187). The cost of nutritional supplements ($11 136) represents an estimated additional 11.6% to the cost of the drugs for these children (Table 4).

**Table 4:** Estimated cost of supplying first-line ART by mean weight for age for 26 weeks

			Estimated cost of 26-weeks ART per child				
				
Age group (years)	Mean weight (kg)	Number of children (251)	ABC	3TC	LPV/r	EFV	Estimated total cost for age group (ZAR)
≤3	9.8	92	907	767	667	N/A	215 372
>3	21.6	159	2274	304	N/A	757	530 265
Estimated total cost for cohort							ZAR 745,637

Conversion rate ZAR to USD using 2010 mid-year exchange rate (02 July 2010: 1ZAR = 0.129USD).

## Discussion

This study investigates the financial costs of providing nutritional supplementation to a rural cohort of HIV-infected children in a public health HIV treatment programme using standardised international guidelines, with costs based on the nutritional supplements available and used in the country. Applying these guidelines, we found that almost half the children initiated on ART in 2010 would have been eligible for nutritional supplementation, reflecting the levels of poverty and comorbid TB in the area (Houlihan *et al*. 2010b). Whilst the proportion of children found to be undernourished at initiation of ART in this study is similar to that found in previous studies (Bolton-Moore *et al*. 2007), further generalisability of our results may be limited by a lack of similar nutritional supplements and differences in programmatic costs in other settings which were not accounted for in this study.

In our programme, the estimated annual cost of purchasing the recommended quantities of nutritional supplementation for 26 weeks for all eligible children initiated during the study period was approximately $11 136. This represents 11.6% of the cost of procuring ART for 26 weeks for *all* children initiating ART during 2010. Nutritional supplements are provided until exit criteria are achieved with a 26-week time frame used in this study (see above). Thereafter, with appropriate supplementation, follow-up and the initiation of ART, children should remain adequately nourished and only require an additional 10% daily energy requirement (NCP-A), which is provided at the household level. Thus, supplementation should only be required once for a maximum period of 26 weeks during a 52-week (1-year) period, whereas ART is required for the entire period. The proportional costs of supplementation in this ART programme for a 1-year period equates to 5.8% of ART costs.

The estimation of costs for purchasing nutritional supplementation will be affected by a number of factors. The proportion of children who would have been eligible for supplements is likely to have been underestimated for three reasons. First, our inability to assign a NCP to 21 children because of missing documentation of clinical staging or prevalent TB may have led to an underestimation of the *number* of children requiring supplementation. Second, as heights or MUACs were not recorded at initiation for the majority of children, we depended on WAZ to estimate undernutrition, which may have resulted in misclassification of some children. Finally, accurate documentation of comorbidities and clinical signs indicative of SAM may have been missing in the case files resulting in an underestimation of the *extent* of undernutrition and inaccurate assignment of children to a NCP.

Difficulties in the accurate recording and interpretation of anthropometric measures by field staff in resource-poor settings have been found to range from a lack of confidence in calculating accurate age from birth dates (Hamer *et al*. 2004), difficulties in plotting growth charts appropriately (resulting in inaccurate WAZ and WHZ) (Qayad 2005), and limited access to, and training in, the use of reference growth charts and equipment such as scales and stadiometers (Duggan 2010). These issues are likely to have been present within the Hlabisa HIV Programme and to have affected the accurate detection of children who were undernourished and required supplementation during the study period.

It is also possible that an overestimation of costs *per child* may have occurred when using the 26-weeks time frame for the supply of supplementation. In practice, the actual time taken to nutritional recovery defined by the exit criteria is often <26 weeks (except for those with concurrent TB who require supplementation until the completion of their TB treatment). Clinically, we found that children with moderate malnutrition often achieved exit criteria, and moved to NCP-A, by approximately 12 weeks and those with SAM by 12–16 weeks. Assessing each child carefully and exiting them from additional supplementation appropriately would reduce supply costs to a total of ZAR57,079 ($7363) (ZAR20,960 for the management of children requiring 12-week NCP-B only, and ZAR36,119 for those requiring NCP-C initially (10-weeks Sibusiso and 6-weeks Future Life Porridge)). This reduces the proportional costs further to 7.6% of ART costs for 26 weeks and approximately 3.8% over the course of 52 weeks.

Alternatively, if we assume that the 48 children eligible for NCP-B who did not have prevalent TB at diagnosis required only 13-weeks NCP-B instead of 26 weeks (and continued to provide 26-week NCP-B to those with prevalent TB), the cost of supplying NCP-B would be reduced to ZAR34,072 ($4395). Furthermore, if we assume that children with SAM recover more quickly than the 10 weeks allowed for here and require only 6 weeks of NCP-C followed by 20-week NCP-B, the costs of supplying nutritional supplements to this groups is reduced to ZAR29,528 ($3809). Combining these two estimations would reduce the overall cost of supplementation for all children to ZAR63,600 ($8204), which would represent 4.2% of the cost of 52-weeks ART to the same group.

This analysis is limited to the costs of purchasing supplements and compares these to purchase costs of ART. It does not account for the costs of staff training, transport, distribution and wastage of goods, inpatient care, outpatient review, and opportunity costs for carers to attend clinics. Further analyses are required to estimate these additional costs, which may impact on the feasibility of adopting the Guidelines.

The provision of nutritional supplements by the Programme during, and prior to, 2010 was relatively unstructured and relied on physicians or nurses identifying undernourished children. Studies in HIV-uninfected children have found improvements in anthropometric status and clinical outcomes, including greater rates of weight gain and reduced nutritional relapse and mortality, following the implementation of formalised nutritional supplementation with RTUF (Weisstaub & Araya 2008) even when compared with traditional milk and flour supplementation (Ciliberto *et al*. 2005; Bhutta *et al*. 2008). It is possible that, in the absence of Guidelines, undernourished children or those with comorbidities would not receive supplementation in the correct amounts for a prescribed period of time.

Good nutritional management is essential for the improvement of nutritional outcomes and anthropometric measures in HIV-infected children (Kim *et al*. 2012), but especially important for those who are undernourished or have additional comorbidities (including tuberculosis) to reduce their higher burden of morbidity and mortality (Hesseling *et al*. 2005). Assuming the level of undernutrition is representative of other paediatric populations initiating ART in resource-poor regions, this additional cost should be budgeted for in programmes.

Undernourished children and those with comorbidities represent an extremely vulnerable group of HIV-infected children. This study illustrates the low proportional cost of procuring nutritional supplements for HIV-infected children in resource-poor areas. The provision of nutritional supplements should be considered for the optimal management of these children to improve their poor outcomes. We recommend that programmes explore the integration of nutritional care for HIV-infected children along with the provision of ART either by providing supplements themselves or through partnership with organisations that have the resources to provide these.

## References

[b1] Barnighausen T, Tanser F, Gqwede Z, Mbizana C, Herbst K, Newell ML (2008). High HIV incidence in a community with high HIV prevalence in rural South Africa: findings from a prospective population-based study. AIDS.

[b2] Bhutta ZA, Ahmed T, Black RE (2008). What works? Interventions for maternal and child undernutrition and survival. Lancet.

[b3] Black RE, Allen LH, Bhutta ZA (2008). Maternal and child undernutrition: global and regional exposures and health consequences. Lancet.

[b4] Bolton-Moore C, Mubiana-Mbewe M, Cantrell RA (2007). Clinical outcomes and CD4 cell response in children receiving antiretroviral therapy at primary health care facilities in Zambia. JAMA.

[b5] Ciliberto MA, Sandige H, Ndekha MJ (2005). Comparison of home-based therapy with ready-to-use therapeutic food with standard therapy in the treatment of malnourished Malawian children: a controlled, clinical effectiveness trial. American Journal of Clinical Nutrition.

[b6] Davies MA, Keiser O, Technau K (2009). Outcomes of the South African National Antiretroviral Treatment Programme for children: the IeDEA Southern Africa collaboration. South African Medical Journal.

[b7] Duggan MB (2010). Anthropometry as a tool for measuring malnutrition: impact of the new WHO growth standards and reference. Annals of Tropical Paediatrics.

[b8] Fergusson P, Chinkhumba J, Grijalva-Eternod C, Banda T, Mkangama C, Tomkins A (2009). Nutritional recovery in HIV-infected and HIV-uninfected children with severe acute malnutrition. Archives of Disease in Childhood.

[b9] Hamer C, Kvatum K, Jeffries D, Allen S (2004). Detection of severe protein-energy malnutrition by nurses in The Gambia. Archives of Disease in Childhood.

[b10] Hesseling AC, Westra AE, Werschkull H (2005). Outcome of HIV infected children with culture confirmed tuberculosis. Archives of Disease in Childhood.

[b11] Houlihan CF, Bland RM, Mutevedzi PC (2010a). Cohort profile: Hlabisa HIV treatment and care programme. International Journal of Epidemiology.

[b12] Houlihan CF, Mutevedzi PC, Lessells RJ, Cooke GS, Tanser FC, Newell ML (2010b). The tuberculosis challenge in a rural South African HIV programme. BMC Infectious Diseases.

[b13] Kim MH, Cox C, Dave A (2012). Prompt initiation of ART With therapeutic food is associated with improved outcomes in HIV-infected Malawian children with malnutrition. Journal of Acquired Immune Deficiency Syndromes.

[b14] Manary MJ, Ndkeha MJ, Ashorn P, Maleta K, Briend A (2004). Home based therapy for severe malnutrition with ready-to-use food. Archives of Disease in Childhood.

[b15] Muhawava W (2008). Trends in Economic Status of Households in the ACDIS: Monograph Series Number 3.

[b17] Qayad MG (2005). Competence of maternal and child health clinic workers in detecting malnutrition in Somalia. African Health Sciences.

[b16] South African Dept of Health (2010). Guidelines for the Management of HIV in Children.

[b18] Sutcliffe CG, van Dijk JH, Bolton C, Persaud D, Moss WJ (2008). Effectiveness of antiretroviral therapy among HIV-infected children in sub-Saharan Africa. The Lancet Infectious Diseases.

[b19] Sutcliffe CG, van Dijk JH, Munsanje B (2011). Weight and height z-scores improve after initiating ART among HIV-infected children in rural Zambia: a cohort study. BMC Infectious Diseases.

[b20] Wallrauch C, Heller T, Lessells R, Kekana M, Barnighausen T, Newell ML (2008). High uptake of HIV testing for tuberculosis patients in an integrated primary health care HIV/TB programme in rural KwaZulu-Natal. South African Medical Journal.

[b21] Weisstaub G, Araya M (2008). Acute malnutrition in Latin America: the challenge of ending avoidable deaths. Journal of Pediatric Gastroenterology and Nutrition.

[b22] WHO (1986). Use and interpretation of anthropometric indicators of nutritional status. Bulletin of the World Health Organisation.

[b23] WHO (1999). Management of Severe Malnutrition: A Manual for Physicians and Other Senior Health Workers.

[b24] WHO (2006). https://www.who.int/entity/hiv/pub/guidelines/art/en/index.html.

[b25] WHO (2009). Guidelines for an Integrated Approach to Nutritional Care of HIV-Infected Children (6 month–14 years).

[b27] World Health Organization, United Nations Children's Fund (2009). http://www.who.int/nutrition/publications/severemalnutrition/9789241598163/en/index.html.

